# Adenomyomatosis of distal common bile duct: A case report and systemic review

**DOI:** 10.1097/MD.0000000000041649

**Published:** 2025-02-28

**Authors:** Chong-You Weng, Cheng-Hung Lee

**Affiliations:** a Division of Surgical Intensive Care Unit, Buddhist Tzu Chi Medical Foundation Dalin Tzu Chi Hospital, Chia Yi, Taiwan; b Department of General Surgery, Buddhist Tzu Chi Medical Foundation Dalin Tzu Chi Hospital, Chia Yi, Taiwan; c School of Medicine, Tzu Chi University, Hualien City, Taiwan.

**Keywords:** adenomyomatosis, common bile duct, Whipple operation

## Abstract

**Rationale:**

Adenomyomatosis is a rare benign condition characterized by epithelial proliferation and diverticular formation in the gastrointestinal tract, most commonly affecting the gallbladder. Its occurrence in the bile duct is uncommon but significant, as it can cause biliary obstruction, cholestasis, and abdominal pain—symptoms that closely mimic early-stage cholangiocarcinoma. This overlap creates diagnostic challenges and increases the risk of misdiagnosis and overtreatment, making accurate identification essential.

**Patient concerns:**

A 54-year-old woman presented with persistent epigastric dull pain. Imaging studies revealed a dilated common bile duct (CBD), raising concerns about biliary pathology.

**Diagnoses:**

Abdominal computed tomography demonstrated a contrast-enhanced 1.6 cm tumor-like mass in the distal CBD, leading to a presumptive diagnosis of malignancy. Laboratory findings showed an elevated γ-glutamyl transferase level, while other tumor markers were within normal ranges.

**Interventions:**

Due to the high suspicion of malignancy, the patient underwent a Whipple procedure for both diagnostic and therapeutic purposes.

**Outcomes:**

Histological examination of the resected specimen surprisingly revealed adenomyomatosis of the CBD, confirming a benign diagnosis. The patient had an uneventful postoperative recovery and was discharged 19 days after surgery.

**Lessons:**

This case highlights the diagnostic challenge posed by adenomyomatosis of the distal CBD, as its radiological appearance can closely resemble malignancy. Clinicians should be aware of this rare entity to avoid unnecessary aggressive surgical interventions.

## 1. Introduction

Adenomyomatosis, a benign lesion characterized by epithelial proliferation and diverticular formation in the gastrointestinal tract, most commonly occurs in the gallbladder.^[1]^ Although gallbladder adenomyomatosis is detected in 2.0% to 8.7% of cholecystectomy specimens from adults, adenomyomatosis in the biliary tree is infrequent.^[2]^ While adenomyomatosis within the gallbladder often presents asymptomatically,^[3]^ its occurrence in the bile duct may result in biliary obstruction, cholestasis, and abdominal pain.^[4]^ The pathogenesis of adenomyomatosis remains unclear. We report a patient presented with persistent dull epigastralgia, a tumor-like mass in the distal common bile duct (CBD) in computed tomography (CT) images, and underwent a Whipple procedure. Adenomyomatosis was confirmed upon histopathological diagnosis.

## 2. Case report

**Figure 1.**
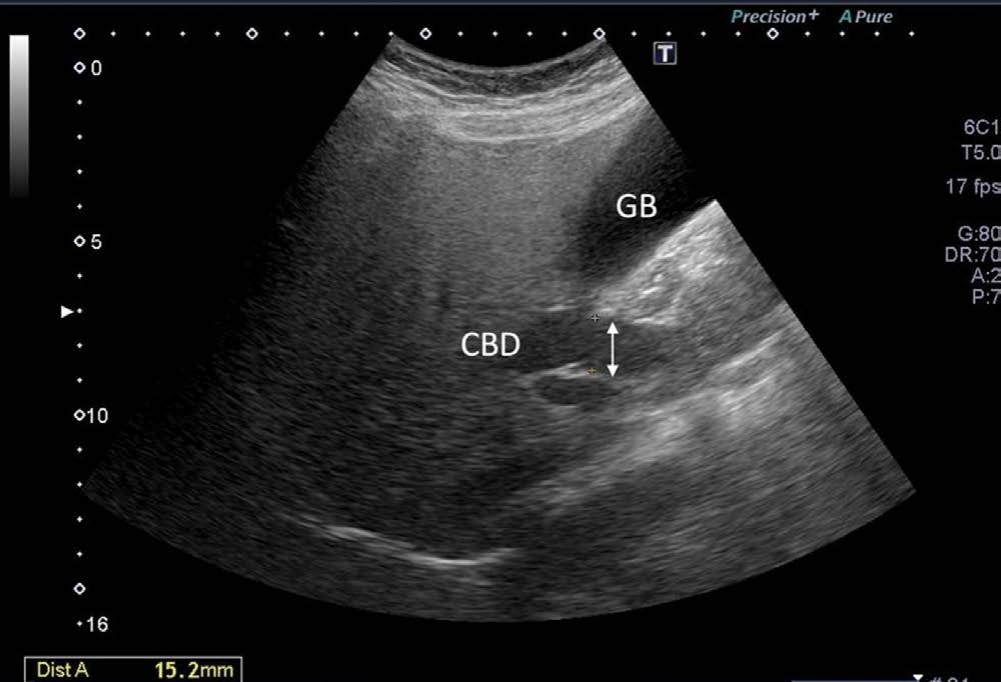
Abdominal ultrasonography showed dilated common bile duct up to 15.2 mm without obvious stones nor mass.

**Figure 2.**
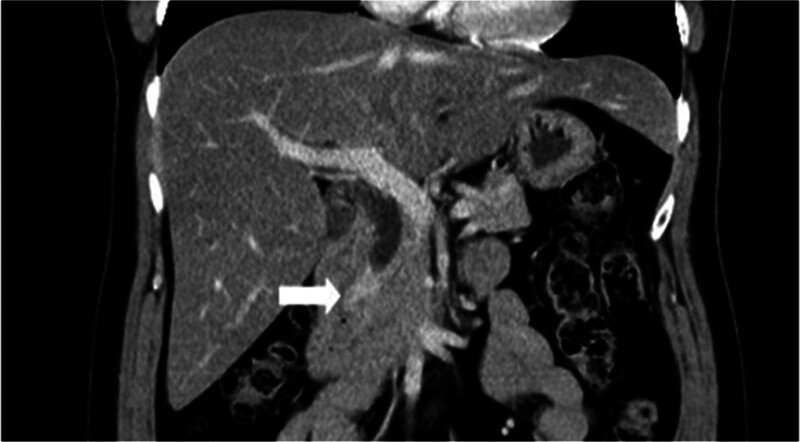
CT showed enhanced thick wall and tumor-like mass about 1.6 cm over distal common bile duct (arrow). CT = computed tomography.

**Figure 3.**
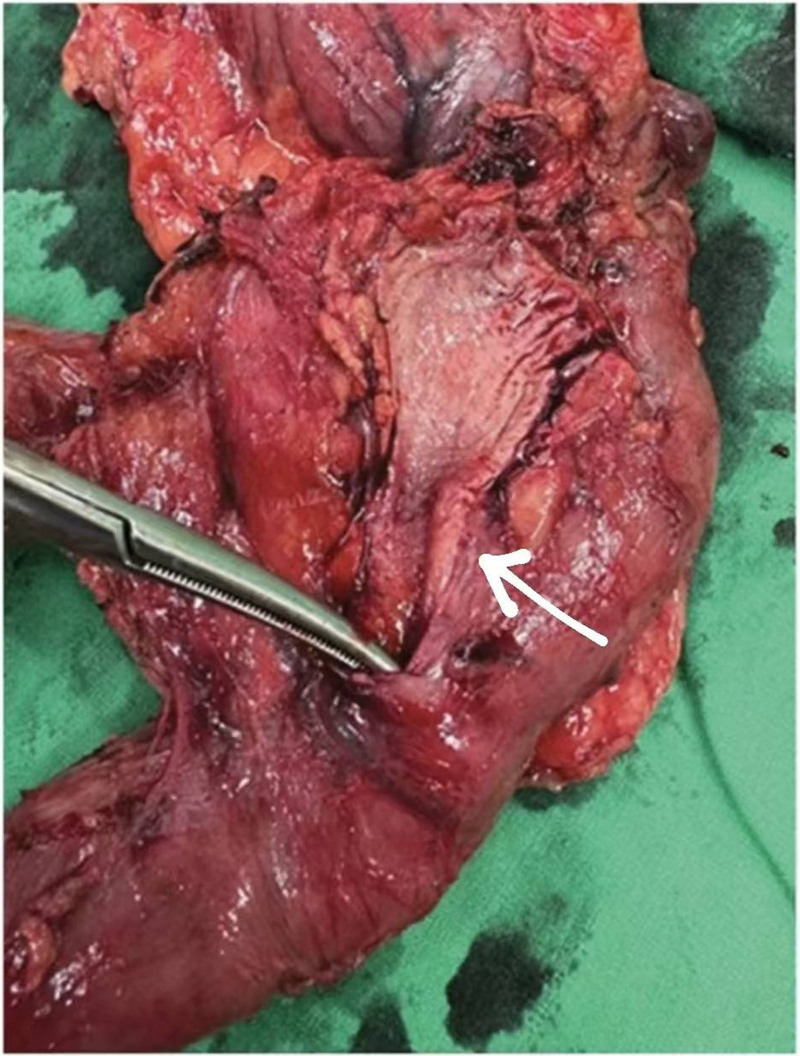
Tissue resected from Whipple operation. Posterior view of common bile duct (CBD). Distal CBD wall thickness (arrow). The tip of instrument is passed from distal CBD and ampullar vater into 2nd portion of duodenum.

**Figure 4.**
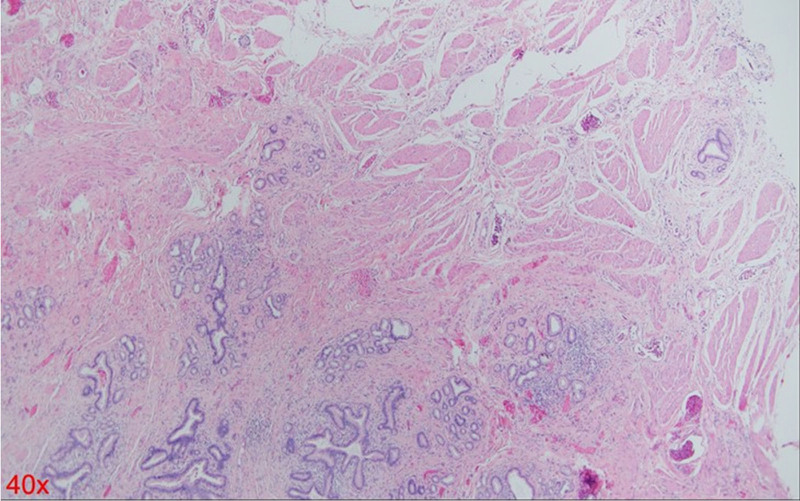
H&E histology (40×). H&E = Hematoxylin and Eosin.

A 54-year-old woman with a medical history of hypertension and hyperlipidemia presented to the outpatient department complaining of persistent epigastric dull pain. Abdominal ultrasonography revealed significant dilation of the CBD (up to 15.2 mm); however, no obvious intraductal mass, bile duct wall thickening, CBD stones, or pancreatic head tumors were identified (Fig. 1). Subsequent abdominal CT demonstrated a contrast-enhanced tumor-like mass measuring approximately 1.6 cm in the distal CBD, characterized by wall thickening without distinct intraductal lesions. Magnetic resonance imaging (MRI) confirmed narrowing of the distal CBD with mild upstream dilatation measuring 10.9 mm, without liver, pancreatic, or lymph node involvement (Fig. 2). Physical examination of the abdomen revealed no tenderness, and the patient denied experiencing fever, nausea, vomiting, or loss of appetite. Laboratory investigations revealed an elevated level of γ-glutamyl transferase (GGT) at 130 U/L, while regular blood tests, as well as levels of carcinoembryonic antigen (CEA), cancer antigen 153 (CA153), and CA199, were within normal ranges (Table 1). Suspecting a bile duct tumor, a Whipple procedure was performed for both diagnostic and definitive treatment purposes (Fig. 3). Histological examination of CBD sections revealed adenomyomatous hyperplasia characterized by clusters of benign biliary glands embedded within fibromuscular stroma and surrounded by lymphoplasmacytic inflammatory cells (Fig. 4). The lesion exhibited no signs of malignancy, confirming its benign nature. The patient experienced a satisfactory recovery and was discharged 19 days post-surgery.

**Table 1:** Laboratory examination.

Parameter	Value
Hemoglobin, g/dL White blood cells, ×10^9^/L	13.1 4450
Platelets, ×10^9^/L Mean corpuscular volume, fL Mean platelet volume, fL Total bilirubin, mg/dL Bilirubin direct, mg/dL AST, IU/ ALT, IU/L GGT, IU/L Amylase, IU/L Monocyte, % CEA, ng/mL CA153, U/mL CA199, U/mL	314,000 84.6 9.0 0.28 0.08 20 26 130 31 9.0 0.24 7.59 18.65

ALT = alanine aminotransferase, AST = aspartate aminotransferase, GGT = γ-glutamyl transferase, CEA = carcinoembryonic antigen.

## 3. Discussion

This case presents a unique aspect in which preoperative CT imaging revealed distal CBD dilation and a contrast-enhanced tumor-like mass, a presentation typically indicative of malignancy. However, the final pathology report confirmed a benign diagnosis. This scenario contributes valuable insight into the potential for benign etiologies in cases presenting with similar radiological features, thereby enhancing diagnostic considerations in clinical practice. Preoperatively, abdominal ultrasonography revealed significant dilation of the CBD. However, no obvious findings of intraductal tumors, bile duct wall thickening, CBD stones, or pancreatic head tumors were observed. Suspecting distal bile duct obstruction, an abdominal CT scan was performed, which confirmed the presence of a distal CBD tumor. An MRI was subsequently performed for confirmation. Given the strong suspicion of malignancy based on CT and MRI findings, endoscopic ultrasonography (EUS) was not performed, and the decision was made to proceed directly with surgery. During surgery, the lesion was identified as thickening of the distal CBD wall within the pancreatic head. This anatomical location explains why preoperative abdominal ultrasonography failed to detect the lesion, revealing only dilated bile ducts. If EUS had been performed preoperatively, it might have visualized the stenotic portion of the distal CBD in the pancreatic head, but it cannot reliably distinguish whether the cause is a malignant tumor, or a choledocholithiasis.

Adenomyomatosis, also called adenomyomatous hyperplasia, adenomyosis, or adenomyoma, is a rare benign lesion primarily found in the gallbladder. Although typically confined to the gallbladder, cases have been sporadically documented in the stomach, small bowel, bile ducts, and ampulla of Vater. For patients with adenomyomatosis outside of gallbladder, 12.5% patients in left hepatic duct, 12.5% in common hepatic duct, 34.4% in CBD, and 37.5% in ampulla of Vater.^[5]^ Adenomyomatosis in the ampullary region and CBD can mimic malignant behavior, often leading to biliary tract obstruction and prompting aggressive treatments like Whipple procedure. Despite its benign nature in most instances, the clinical implications of adenomyomatosis in these sites necessitate careful management due to potential severe complications.

Adenomyomatosis of the distal CBD can mimic malignancy, posing diagnostic challenges. Several case reports have highlighted the difficulty in distinguishing adenomyomatosis from cancer in the distal CBD, as the clinical presentations and radiological findings can be similar. Xu et al^[6]^ described a 68-year-old woman who exhibited significant liver enzyme elevations and a dilated CBD. A firm mass in the distal CBD was discovered during a Whipple procedure and subsequent histological examination confirmed the diagnosis of diffuse adenomyomatous hyperplasia. Similarly, Chandler et al^[7]^ reported a case of a 63-year-old male who presented with lethargy, fever, weight loss, painless jaundice, and a dilated distal CBD. After undergoing a Whipple procedure, adenomyomatous hyperplasia was confirmed.

These cases, along with other reports,^[8–11]^ highlight the minimal differences between adenomyomatosis and cancer in the distal CBD, both in terms of clinical presentation and radiological findings. These studies suggest that adenomyomatosis in the distal CBD can lead to a range of clinical syndromes and varying degrees of liver damage. Consequently, there is a need to focus efforts on identifying precise markers or diagnostic tools to distinguish adenomyomatosis from malignancy in the distal CBD, as accurate diagnosis is crucial for appropriate treatment and management.

Tumors growing in the CBD can cause bile duct obstruction. In addition to the commonly used CT and MRI scans, the most widely developed diagnostic modality is endoscopic retrograde cholangiopancreatography (ERCP). ERCP allows for tissue sampling through various approaches via the ampulla of Vater, providing more accurate preoperative guidance. However, this procedure has limitations in terms of sensitivity. Despite continuous advancements in equipment and high reported success rates in the literature, clinical practice often presents a dilemma where imaging studies suggest a tumor, but ERCP biopsies reveal only benign hyperplasia. This situation makes it challenging to differentiate between benign conditions such as adenomatous narrowing or cholangitis and malignant tumors. Ultimately, surgical resection frequently confirms the presence of a malignant tumor.

In this patient’s case, preoperative imaging clearly identified a tumor, and both CT and MRI scans strongly suggested cancer. The primary finding was significant wall thickening of the bile duct without a large mass formation. Consequently, ERCP biopsy would likely have difficulty capturing the tumor tissue, potentially leading to a false-negative report of benign changes. Regardless of the biopsy result, surgery would still be necessary due to the high suspicion of malignancy based on imaging findings. Therefore, this patient did not undergo preoperative ERCP. If the patient had undergone ERCP with tissue sampling from the correct location, the histological findings would likely resemble those shown in Figure 4, demonstrating adenomatous hyperplasia. This scenario would present a clinical dilemma, where imaging suggests a tumor suspicious for cancer, but the biopsy indicates a benign process

A PubMed search employing the keywords “adenomyomatous hyperplasia” or “adenomyoma” or “adenomyomatosis” in conjunction with “common bile duct” excluding “cystic duct,” “ampulla of Vater,” and “pancreatic duct,” yielded 21 case reports in English. Following further screening to exclude references unrelated to adenomyomatosis treatment and limited to the intrahepatic bile duct, a total of 14 case reports encompassing 15 patients were identified and summarized in Table 2.^[6,7,10,12–22]^ Among the selected case reports, two-third of the patients were female (n = 10, 66.7%). The age range of the patients spanned from 31 to 74 years with a mean age of 59.7 years. Among these, 9 patients (60%) presented with jaundice, 7 (46.7%) reported abdominal pain, 4 (26.7%) exhibited fever. Additionally, 1 patient (6.7%) each reported experiencing nausea, vomiting, loss of appetite, and weight loss. Preoperative biochemical analysis revealed that 3 patients (20%) exhibited normal biochemical parameters, 7 (46.7%) had elevated bilirubin levels, 6 (40%) displayed increased alkaline phosphatase (ALP) levels, 5 (33.3%) had elevated alanine aminotransferase (ALT) levels, 4 (26.7%) exhibited increased aspartate transaminase (AST) levels, and 3 (20%) showed elevated GGT levels. Imaging diagnoses indicated bile duct dilation in 9 patients (60%) and bile duct stenosis in 4 (26.7%). Surgical interventions included Whipple operation in 9 patients (60%), local resection in 4 (26.7%), and resection with biopsy forceps in 1 patient (6.7%). Preoperative biopsy was performed in 4 patients (26.7%) and all of them were reported benign or no malignant cell findings. Even in this situation, 2 patients still treat it as cancer and undergo Whipple operation.

**Table 2:** Cases of adenomyomas of the common bile duct.

Reference	Age, sex	Chief complaint	Laboratory changes	Imaging changes	Preoperative biopsy	Surgery
Ikei et al, 1989^[12]^	71, F	Jaundice, fever, abdominal pain	↑ALP, AST, ALT, and conjugated bilirubin	BD dilation in CT	Not performed	Whipple
	52, F	Abdominal pain	Normal	CBD dilation and a spherical shadow defect at the end of BD in ERCP	Not performed	Whipple
Legakis et al, 1990^[13]^	55, F	Jaundice and abdominal pain	↑ALP and conjugated bilirubine	Not described	Not performed	Local resection
Läuffer et al, 1998^[14]^	69, F	Abdominal pain	Normal	Calculi in the gallbladder and dilated extrahepatic bile ducts	Suggestive of adenoma	Local resection
Tsukamoto et al, 1999^[15]^	31, F	Epigastroalgia	Cholestasis	CBD stenosis	Not performed	Local resection
Ojima et al, 2000^[10]^	64, F	Jaundice, fever, appetite loss, and abdominal pain	Not performed	Possible stenosis in distal CBD in ERCP	Not performed	Whipple
Shu et al, 2008^[16]^	51, M	Jaundice, nausea, and weight loss	↑ALT, AST, ALP, GGT, and total bilirubine	Dilated BD and pancreatic duct in MRI and MRCP; CBD distension but stricture at pancreatic duct in US	Not performed	Whipple
Iwaki et al, 2008^[17]^	62, F	Jaundice	↑ALP	Thickening wall of lower CBD; lower CBD stenosis in ERCP	No malignant cells detected by cytology	Whipple
Genevay et al, 2009^[18]^	73, M	Jaundice	↑bilirubin and ALP	Dilated BD in CT	Not performed	Whipple and cholecystectomy
Choi et al, 2016^[19]^	42, M	Jaundice, epigastoalgia, and vomiting	↑ALP, ALT, AST, GGT, and conjugated bilirubine	Stricture in CBD in CT	No evidence of malignant cells by biopsies	Whipple
D’Assuncao et al, 2016^[20]^	50, F	Abdominal pain	Normal liver tests	Dilated CBD in MRI; a hypoechoic lesion of 5.2 mm near AV in EUS	Not performed	Endoscopic resection with biopsy forceps
Chandler et al, 2018^[7]^	63, M	Jaundice, lethargy, fever, and weight loss	↑Total bilirubin	Distal CBD dilation in US	Nondiagnostic	Whipple
Xu et al, 2019^[6]^	68, F	Abdominal pain	↑ GGT; ↓ AST	Proximal BD dilation in MRCP; enhanced wall of BD in CT and MRI; distal CBD obstruction in EUS	Not performed	Local resection
Gouveia et al, 2021^[21]^	70, F	Epigastralgia	↑AST and ALT	CBD dilation in CT; CBD dilation with a localized stenosis 1 cm above the AV in MRCP; dilated CBD and a poorly defined hypoechogenic mass in distal CBD in EUS	Not performed	Whipple
Tan et al, 2022^[22]^	74, M	Fever and jaundice	↑serum bilirubin, ALP, ALT	calculus in distal common bile duct by CT	Columnar-lined epithelium with no features of high-grade dysplasia or malignancy.	Cholecystectomy with open CBD excision

ALP = alkaline phosphatase, ALT = alanine aminotransferase, AST = aspartate transaminase, AV = ampulla of Vater, BD = bile duct, CBD = common bile duct, CT = computed tomography, ERCP = endoscopic retrograde cholangiopancreatography, EUS = endoscopic ultra sound, MRCP = magnetic resonance cholangiopancreatography, MRI = magnetic resonance imaging.

Adenomyosis can occur at any location along the biliary tract, with the gallbladder being the most common site. In cases of gallbladder adenomyosis, a characteristic finding is the presence of diffuse gallbladder wall thickening, which can be readily identified on ultrasound examination. Typically, thickened gallbladder walls due to adenomyosis do not cause symptoms. However, when adenomyosis involves the bile ducts and leads to biliary obstruction, it can raise significant clinical suspicion for malignancy.

We reported this rare case to provide clinicians with insights into the diagnosis of a benign tumor that can cause biliary obstruction, like malignancy does. Understanding the diagnostic features of bile duct adenomyosis is crucial to avoid misdiagnosis and unnecessary aggressive treatment.

## 4. Limitation

This report has certain limitations. As a single-case report, the findings may not be generalizable to a broader population. Additionally, the diagnostic challenges in distinguishing adenomyomatosis from malignancy using imaging techniques highlight the need for more advanced diagnostic tools or biomarkers. Furthermore, the absence of preoperative EUS in this case limited our ability to evaluate its potential diagnostic utility.

## 5. Conclusion

We presented a case with adenomyoma, whose abdominal CT images showed dilated CBD and a contrast-enhanced tumor-like mass at distal CBD. The radiographic property of this benign tumor provides additional information of benign diagnosis for similar cases in clinical practice.

## Acknowledgments

Authors thank our pathology department for detailed pathologic information and image.

## Author contributions

**Conceptualization:** Chong-You Weng.

**Data curation:** Chong-You Weng.

**Formal analysis:** Chong-You Weng, Cheng-Hung Lee.

**Project administration:** Chong-You Weng.

**Resources:** Cheng-Hung Lee.

**Software:** Chong-You Weng.

**Supervision:** Cheng-Hung Lee.

**Validation:** Cheng-Hung Lee.

**Visualization:** Cheng-Hung Lee.

**Writing – original draft:** Chong-You Weng, Cheng-Hung Lee.

**Writing – review & editing:** Cheng-Hung Lee.
